# A Novel Method of Magnetic Nanoparticles Functionalized with Anti-Folate Receptor Antibody and Methotrexate for Antibody Mediated Targeted Drug Delivery

**DOI:** 10.3390/molecules27010261

**Published:** 2022-01-01

**Authors:** Madeeha Shahzad Lodhi, Fatima Khalid, Muhammad Tahir Khan, Zahoor Qadir Samra, Shabbir Muhammad, Yu-Juan Zhang, Kejie Mou

**Affiliations:** 1 School of Biochemistry and Biotechnology, University of the Punjab, Lahore 54890, Pakistan; fatimakhalid91@hotmail.com (F.K.); samra201@yahoo.com (Z.Q.S.); 2 Institute of Molecular Biology and Biotechnology, The University of Lahore, Lahore 58810, Pakistan; tahirmicrobiologist@gmail.com; 3 Department of Physics, College of Science, King Khalid University, Abha 61413, Saudi Arabia; mshabbir@kku.edu.sa; 4 College of Life Sciences, Chongqing Normal University, Shapingba, Chongqing 401331, China; zhangyj@cqnu.edu.cn; 5 Department of Neurosurgery, Bishan Hospital of Chongqing, Chongqing 402760, China

**Keywords:** targeted drug delivery, anti-folate receptor antibody, methotrexate, anti-methotrexate, folate receptors, immunotherapy, cancer treatment

## Abstract

Therapeutic effects of anticancer medicines can be improved by targeting the specific receptors on cancer cells. Folate receptor (FR) targeting with antibody (Ab) is an effective tool to deliver anticancer drugs to the cancer cell. In this research project, a novel formulation of targeting drug delivery was designed, and its anticancer effects were analyzed. Folic acid-conjugated magnetic nanoparticles (MNPs) were used for the purification of folate receptors through a novel magnetic affinity purification method. Antibodies against the folate receptors and methotrexate (MTX) were developed and characterized with enzyme-linked immunosorbent assay and Western blot. Targeting nanomedicines (MNP-MTX-FR Ab) were synthesized by engineering the MNP with methotrexate and anti-folate receptor antibody (anti-FR Ab). The cytotoxicity of nanomedicines on HeLa cells was analyzed by calculating the % age cell viability. A fluorescent study was performed with HeLa cells and tumor tissue sections to analyze the binding efficacy and intracellular tracking of synthesized nanomedicines. MNP-MTX-FR Ab demonstrated good cytotoxicity along all the nanocomposites, which confirms that the antibody-coated medicine possesses the potential affinity to destroy cancer cells in the targeted drug delivery process. Immunohistochemical approaches and fluorescent study further confirmed their uptake by FRs on the tumor cells’ surface in antibody-mediated endocytosis. The current approach is a useful addition to targeted drug delivery for better management of cancer therapy along with immunotherapy in the future.

## 1. Introduction

Cancer is a major cause of death worldwide, accounting for 10 million deaths in 2020. The incidence rises dramatically and the overall risk of cellular repair mechanisms is less effective in older age [1], as the balance of cells and effects on the immune system may lead to death [2]. Among therapeutic efforts, nano-biotechnology plays an important role in the therapeutic strategy of targeted drug delivery. It is more specific and targeted than conventional approaches to cancer treatment [3,4,5,6,7]. In conventional therapeutic efforts, the drugs not only destroy the cancer cells but also the normal healthy cells including those of the immune system. Therefore, targeted strategies including active and passive deliveries have been applied to minimize the risk of normal cell destruction [8].

Currently, nano-based approaches for cancer detection and treatment are receiving more attention because of their efficacy in targeted drug delivery [8,9,10]. The surface-to-volume ratio is a distinctive property of nanoparticles (NPs) and is more suitable for surface functionalization. Moreover, NPs can be engineered with therapeutic agents and targeting ligands in targeted drug delivery with better tumor specificity than traditional approaches [11]. Nano-sized material applications provide more accurate methods of treatment, as they affect interactions at the cellular and molecular levels [12,13,14,15].

Among different nanoparticles, MNPs have immense application in the different fields of magnetic resonance imaging, magnetic fluids, the controlled release of medicine, information storage, etc. In addition to these applications, MNPs have gained more importance in the field of oncology and nanomedicine [16,17].

Tumor-associated antigens have attracted the attention of scientists for immunotherapy purposes, eradicating tumor cells through the immune system. Folate receptor antibodies offer the advantage to be used for targeting purposes and also to enhance immunological responses against cancer/tumor cells through natural killer cells and macrophages by opsonization [18].

Folate receptors are one of the important targeted sites for drug delivery due to their overexpression on the majority of cancerous tissues, with limited expression on normal cells. Folate receptors are membrane-bound glycoproteins of approximately 37–40 Kda in size that have a nanomolar affinity for folate. Folate internalizes after attachment to folate receptors by receptor-mediated endocytosis [19,20,21,22]. Among the different FRs, FLOR-α and FLOR-β are highly specific and abundantly expressed tumor markers that may be targeted through particular FR ligands [23] as compared to FR FLOR-γ [24].

Numerous anti-cancerous agents are used against various types of cancers. Among these, methotrexate (MTX) is an effective anti-cancer agent that is used for the treatment of various cancers, including those of the breast, bladder, lung, lymphoma, and autoimmune diseases [25,26,27,28,29]. MTX acts as a folate inhibitor and attaches itself to the receptor for obstructing the metabolism of folic acid in cells by blocking dihydrofolate reductase activity [30,31]. The DHFR is more active in converting the reductase of dihydrofolate to tetrahydrofolate [32]. Moreover, folic acid is essential for DNA synthesis; the deficiency of tetrahydrofolate, in turn, reduces the synthesis of thymidine, which finally causes the inhibition of DNA synthesis [33,34,35,36].

Scientists have reported many therapeutic strategies based on folate receptor targeting with nano formulations for targeting and therapeutics against different cancers [37,38,39]. Targeting cancer through anti-FR antibody may be a useful approach in cancer therapy due to targeted antibody-mediated endocytosis and for immunotherapy. The drug nano-composites may increase therapeutic efficiency and reducing cytotoxicity. Further, receptor-specific ligands enhance tissue absorption and efficiency. Receptor and ligand interactions may be considered as the best method for targeting cancerous cells using chemotherapeutic drugs. The highly expressed FR on cancer cells may be targeted through a natural ligand, i.e., folic acid [40]. An antibody drug conjugate comprising folate receptor alpha (FRα) antibody and eribulin as payload was designed to form MORAb-202 ADC. This showed higher tumor suppression and specificity than eribulin alone in breast and non-small lung cancer [41].

In the current research work, anti-folate receptor antibodies were used for targeting folate receptors. Antibodies were produced against purified folate receptors from cancerous cells. Folate receptors were purified using a novel technique of magnetic affinity purification and utilized for the production of antifolate receptor antibody. Folate receptor purification using nanotechnology assures the purification of intact protein in biologically active form. A novel targeting nanomedicine formulation was prepared by conjugation of MNP with MTX and anti-FR antibody for targeted drug delivery through antibody-mediated endocytosis. This novel method of targeted drug delivery through folate receptors not only delivers MTX to the cancer cells but also attracts macrophages and natural killer cells to eradicate cancer/tumor cells through opsonization. Therefore, this study may help to identify the synergistic effect of targeted drug delivery along with immunotherapy in the future.

## 2. Materials and Methods

### 2.1. Synthesis and Characterization of MNPs

**Figure 1:**
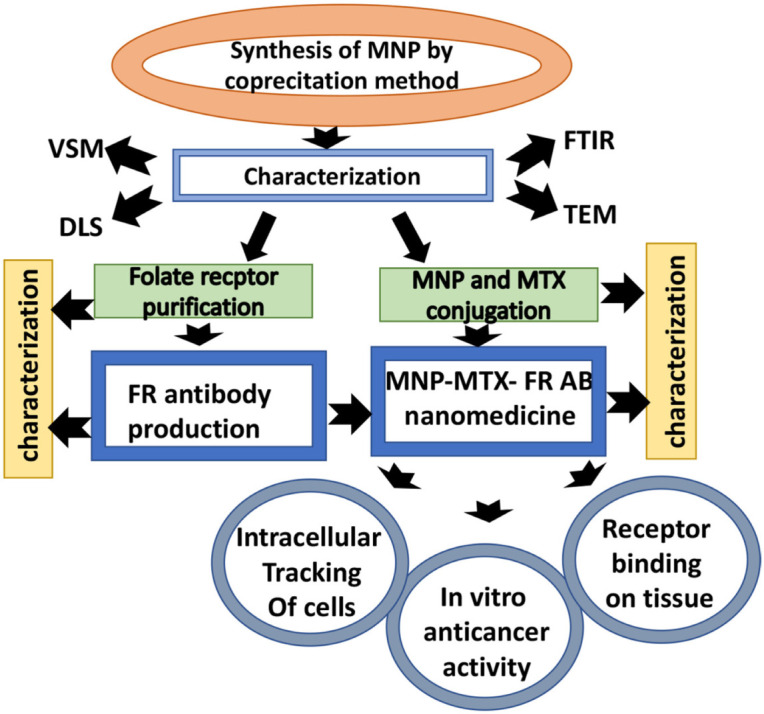
Flow chart of research methodology.

MNPs (iron oxide) were synthesized by coprecipitation as described in the previous study [42]. Briefly, ferrous and ferric chloride solutions were mixed in the ratio of 1:2 at 80 °C for 1 h, and the pH of the solution was adjusted to 10 with NH_4_OH, until black precipitates appeared. Precipitates were collected by filtration, washed with deionized water, and acetone dried. Shape, size, magnetic nature, and surface chemistry of synthesized MNPs were characterized by transmission electron microscopy (TEM) (Transmission Electron Microscope TEM, JEOL JEM-1010), dynamic light scattering analysis (DLS) (HORIBA Scientific, nanoPartica SZ-100), vibrating sample magnetometer (VSM) (Lakeshore Vibrating Sample Magnetometer, VSM) and Fourier transform infrared spectroscopy (FTIR) (Fourier Transform Infrared spectroscope FTIR, Agilent Technology Cary 630), respectively. Amine-terminated MNPs showed attraction toward the magnet and were stored at 25 °C. Figure 1 shows the flow chart of the methodology.

### 2.2. Magnetic Affinity Purification of Folate Receptors

Amine-terminated fine MNPs were conjugated with the carboxylic group of folic acid using the ethyl carbodiimide (ECD) method for purification of FRs. About 200 mg of folic acid was dissolved in 50 mL of deionized water, and the pH of 8.0 was adjusted using 1.0 M NaOH. Then, 50 mg of MNP suspension was mixed with folic acid solution and sonicated for 10 min at 4 °C on ice. The mixture was warmed for 15 min at 37 °C and mixed with 2 mL of 600 mg 1-ethyl-3-(3-dimethyl aminopropyl)–carbodiimide HCl/mL, slowly added. A pH of 6.0 was achieved with 1.0 M hydrochloric acid, with constant stirring (for 6 h at 25 °C) followed by addition of ECD (400 mg) at 25 °C in the dark. The folic acid-MNP (FA-MNP) conjugates were separated using an external magnetic field. The particles were washed with 50 mL deionized water at room temperature, air-dried, and processed for FTIR analysis [43].

After attachment of folate acid with MNP, HeLa cells were cultured in DMEM in a humidified chamber (5% CO_2_) at 37 °C (complete culturing medium containing 10% FBS, 1X Penicillin/Streptomycin solution (Thermo Scientific Cat#15140122, New York, NY, USA) in DMEM high glucose (Thermo Scientific Cat#12800017)). The growing cells were trypsinized and centrifuged at 3000 rpm for 5 min at 25 °C. HeLa cell (5 gm) pellets were suspended in 2 mL PBS, with a pH of 7.4 and homogenized with 2.5 volumes of 0.014 M KPO_4_ buffer, with a pH of 7.5 on ice. Again, 2 volumes of 0.01 M KPO_4_ buffer, with a pH of 7.5, containing 1.5% Triton X-100, were added in homogenized suspension. The mixture was stirred gently overnight at 4 °C. The suspension was centrifuged at 10,000 rpm at 4 °C for 20 min, and the supernatant was separated. About 100 mg of FA-MNP conjugates was mixed with the supernatant and kept for 1 h under mild shaking at 4 °C for binding of the receptor with folic acid. All purification steps were carried out at 4 °C. One-fourth volume of extraction buffer (0.01 M KPO_4_ with a pH of 7.5, 1 M NaCl, and 1% Triton X-100) was added for 30 min on ice. A magnetic field was applied, and supernatant was removed. Again, one-fourth volume of extraction buffer was added for 30 min with slight agitation, and magnetic separation was performed. The receptor bound to the conjugates was suspended in 2 mL suspension buffer (0.01 M sodium acetate–acetic acid buffer, with a pH of 4.5, containing 1% Triton X-100), including 1 mM PMSF and 0.01% aprotinin. The extracted receptor proteins were dialyzed against 1 X PBS for 6 h at 4 °C. Purified protein was stored at −20 °C after the addition of protease inhibitor 0.1 mM phenylmethylsulfonyl fluoride (PMSF). The dialyzed protein samples were run on 10% preparative SDS gel electrophoresis. The required protein band of 37 kDa was cut and electro-eluted (Harlow and Lane). The purified protein band was again run on 10% SDS-PAGE to check the homogeneity of the protein band. Concentration of the protein was determined by Bradford assay at 595 nm using bovine serum albumin as standard.

### 2.3. Production and Characterization of Rabbit-Anti Folate Receptor Antibody

All animal studies were approved by the Animal Ethics Committee of the faculty of Life Sciences, University of the Punjab, Lahore, Pakistan. Two New Zealand white rabbits of age 3 months were used for immunization to develop anti-folate receptor antibodies. Before starting the immunization process, preimmune blood was drawn from marginal ear vein, to be used as control. The purified FR (50–80 μg/mL PBS) was mixed with an equal volume of Freund’s complete reagent (1:1 ratio) and injected into the rabbits subcutaneously. Booster injections were given for 5 weeks after a 2-week interval. The same concentration was used in booster injection, utilizing incomplete Freund’s adjuvants. The immunized blood was drawn, and the serum was used in ELISA to confirm the development of anti-FR antibodies. The rabbit anti-folate receptor antibodies were purified as described (Thermo scientific, catalog no: 34028) [44].

#### 2.3.1. Characterization of Anti-Folate Receptor Antibodies

##### ELISA for Anti-Folate Receptor Antibody

The purified FR (2 μg/100 μL) and HeLa cells (1 × 10^5^/0.1 mL) were coated separately in triplicate in 24-well ELISA plates followed by incubation for 2 h at 25 °C. Wells were then blocked with 5% skimmed milk in TBS for 1 h at room temperature. Wells were washed with 1X TBS, and rabbit-anti Ab (FR) antibody (1:50 dilution) was added into each well. The plates were incubated for 1 h at 37 °C. After washing with 1X TBS, 100 µL of goat anti-rabbit IgG horse radish peroxidase (HRP) conjugated antibody was added (1:5000 dilution), followed by humidified chamber incubation (1 h at 37 °C). The color was developed after washing with 1X TBS and observed using tetramethylbenzidine (TMB) and H_2_O_2_ as substrate.

##### Western Blot Analysis

The purified FR was run on SDS PAGE (10%) and transferred onto nitrocellulose membrane using semidry procedure [45]. The presence of protein was confirmed on membrane by Ponceau S strain [46], and the blot was dipped in 5% skimmed milk/TBS for 1 h. After washing, the blot was exposed to rabbit anti-FR antibodies (1:50 dilution) and goat anti-rabbit HRP conjugated antibodies (1:5000 dilution) for 1 h each. The presence of antibodies was detected by 0.1% diaminobenzidine and 0.1% hydrogen peroxide as substrate.

### 2.4. Synthesis of Targeting Nanomedicines MNP-MTX-FR Ab

#### 2.4.1. MTX Conjugation to MNP

Amine-terminated MNPs were conjugated with MTX by carbodiimide method. About 100 mg of MTX was dissolved in 25 mL distilled water (pH of 8.0), followed by mixing 20 mg of amine terminated MNPs with MTX. The mixture was sonicated (10 min at 4 °C on ice) and about 400 mg of EDC HCl was added to the mixture (pH 6.0). The mixture was stirred (6 h at 25 °C) and then 400 mg of EDC was added (15 h in dark). MTX-conjugated nanoparticles (MNPs) were isolated (magnetic separation) and washed with deionized water and acetone, respectively. The conjugates were air-dried, and the particles were processed for FTIR observations. The binding of MTX with MNP was calculated.

#### 2.4.2. Anti-Folate Receptor Conjugation with MNP-MTX

Final conjugation of anti-folate receptor antibodies with MNP-MTX was performed by carbodiimide method. About 200 mg from the MNP-MTX particles was taken and suspended in 5 mL of 0.1 M sodium phosphate buffer (pH of 6.0) and mixed with 200 mg freshly prepared solution (4 mL) of 1-Ethyl-3-(3-dimethylaminopropyl) carbodiimide (EDC). After mixing, 1 mg of anti-FR antibody dissolved in PBS (5 mL) was added drop-wise. The suspension was further gently mixed (2 h at room temperature) followed by washing the conjugated particles 2 times using 1X PBS, then stored (PBS, 0.1% BSA, 0.05% sodium azide) at −4 °C.

### 2.5. Immunocharacterization of Nanoformulations

MTX and anti-FR antibody conjugation on MNPs were confirmed by Mag-LISA. For confirmation of MTX conjugation on MNP, about 100 μL of mouse anti-MTX antibody (1:20 dilution in TBS) was coated in micro well-plate (triplicate) for 1 h at 37 °C or overnight at 4 °C. The supernatant was washed and decanted. The wells were blocked with skimmed milk (5%) and nanocomposite (MNP-MTX) was added and incubated for 2 h at 37 °C. Again, after washing, 100 μL of TMB with H_2_O_2_ as substrate was added and color development observed. After confirmation of MTX conjugation on MNP, FR-Ab was conjugated to MNP-MTX nanocomposites, and again, Mag-LISA was performed to confirm the conjugation of anti-FR Ab on MNP-MTX. Mag-LISA was performed in a similar way as described above, except this time the wells were coated with folate receptor protein.

### 2.6. pH-Dependent Stability of Nanomedicines

The binding stabilities of antibodies and MTX with MNPs were estimated at pH 5.5 and 7.4 in different buffer conditions at 37 °C. An amount of 200 µg of nanocomposites was mixed separately with 2.0 mL of 20 mM phosphate buffer pH 5.5 and 20 mM phosphate buffer pH 7.4 and kept at 37 °C. After 3, 6, and 12-h intervals, vials and nanocomposites were separated. The released MTX and antibodies were checked to measure O.D. at 486 nm for MTX and antibodies by Bradford reagents. The released MTX and antibodies were estimated from standard graph.

### 2.7. In Vitro Anticancer Effects of Nanocomposites

In vitro effects of different concentrations of MTX, MNP-FA, MNP-MTX-FA, and MNP-MTX-FR Ab were examined on HeLa cells. HeLa cells (1 × 10^5^) were seeded in 24-well culture plates along with 0.125, 0.25, 0.5, 1, 2 mg/mL of different concentrations of MTX, MNP-FA, MNP-MTX-FA, and MNP-MTX-FR Ab followed by CO_2_ incubation (overnight 37 °C). Effect was examined by MTT assay [47] in comparison with control (non-treated HeLa cells). HeLa cells (1 × 10^3^) were added in 24-well culture plate and incubated with MNP-MTX-FR Ab (24 h at 37 °C) for MTT assay, followed by 2 h incubation when 10 μL of the MTT reagent was added into each well. Next, 100 μL of SDS solution (10%) was added into the wells for a further 2 h in dark. Absorbance was recorded at 570 nm.

### 2.8. Receptor Binding Assay of MNP-MTX-FR Ab Nanomedicine

#### 2.8.1. Intracellular Tracking of Nanomedicine (HeLa Cells)

HeLa cells (1 × 10^6^) were cultured and mixed with 200 μg composite (MNP-MTX-FR Ab) in humidified incubator (37 °C for 2 h). Cells were harvested (1500 rpm for 15 min), and the pellet was suspended in 100 μL DMEM media. Cells (10–15 µL) placed on albumin coated slides were fixed and covered with 5% skimmed milk to block the non-specific binding site. After washing with TBS, cells were incubated with secondary antibody (goat anti-rabbit Fluorescein Isothiocyanate (FITC) conjugated antibody) (1:5000 dilution) for 45 min. Again, after washing, binding was visualized under fluorescent microscope. The presence of MTX in composite bound to HeLa cells was also checked in another slide with anti-MTX antibody. In second case of MTX, after treating the cells with mouse anti-MTX antibody (Supplementary Data) and washing, the cells were incubated with secondary antibody rabbit anti-mouse Texas red conjugated antibody. Fluorescence was observed under microscope with blue and green excitation filter for FITC and Texas red dye, respectively.

#### 2.8.2. Immunohistochemistry (Binding Efficiency with Tumor Tissue)

The binding of nanomedicine (MNP-MTX-FA Ab) was also checked on a mouse tumor. A mouse mammary tumor was cut into small pieces and fixed with 10% buffered formalin in 20 mM Tris-Cl buffer solution with a pH of 7.5 and stored at 4 °C. The small pieces of tumor tissue were dehydrated in ascending series of sucrose (5%, 10%, 20%, and 30%) in 20 mM Tris-Cl buffer with a pH of 7.5 and embedded in OCT compound at −20 °C and stored at −20 °C. Sections (8–10 μm) were cut on cryostat, placed on albumin-coated slides (two slides), and processed further for binding studies. Conjugate MNP-MTX-FR Ab suspended in TBS (5 mg/mL) was added onto the cut tumor sections on the albumin-coated slides and left for 1 h at room temperature in a humidified chamber to attach with the tumor section. The sections on the slides were further treated with 1% glutaraldehyde in TBS for 30 min and blocked with 5% skimmed milk in TBS for 1 h in a humidified chamber at room temperature. After blocking, for FA Ab tracing, tissues on one slide were treated with goat anti-rabbit FITC conjugated antibody (1:1000 dilution) for 45 min. The slide was washed with TBS and observed under blue excitation filter of microscope with green emission. For MTX tracing, tissue on the second slide was incubated first with anti-MTX antibody and then with rabbit anti-mouse IgG Texas red conjugated antibody (1:1000 dilution). This slide was washed with TBS and observed under green excitation filter of microscope with red emission

## 3. Results

### 3.1. Characterization of MNPs

**Figure 2:**
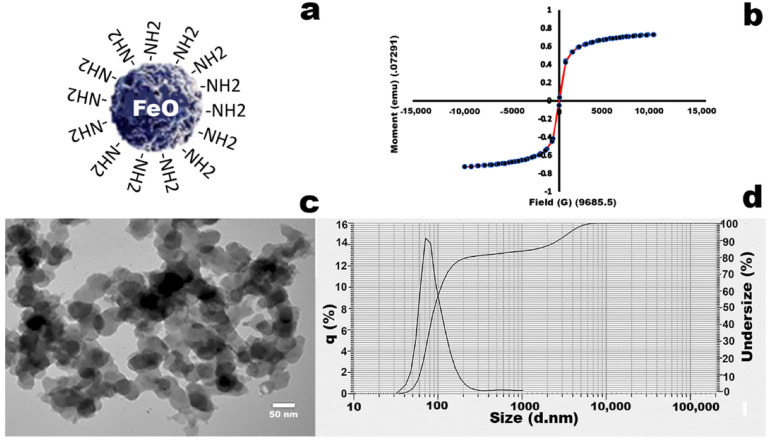
Characterization of MNP. (**a**) MNP structural scheme; (**b**) VSM analysis of MNP. (**c**) shows the TEM results for size and shape. (**d**) DLS analysis shows that the hydrodynamic size distribution of MNP.

MNPs were characterized by VSM, DLS, and transmission electron microscopy (TEM). The superparamagnetic properties of MNPs were confirmed with VSM, showing the hysteresis loop with associated pattern (coercivity (Hci) 47.713G, magnetization (Ms) 0.72923 emu and retentivity (Mr) 33.294 × 10^−3^ emu) (Figure 2b). DLS analysis showed the size of MNPs falling in the range of 50–150 nm (Figure 2d), and TEM observation also confirmed that the shapes of MNPs were irregular spherical, with sizes in the range of 50–150 nm (Figure 2c).

### 3.2. FTIR Analysis

**Figure 3:**
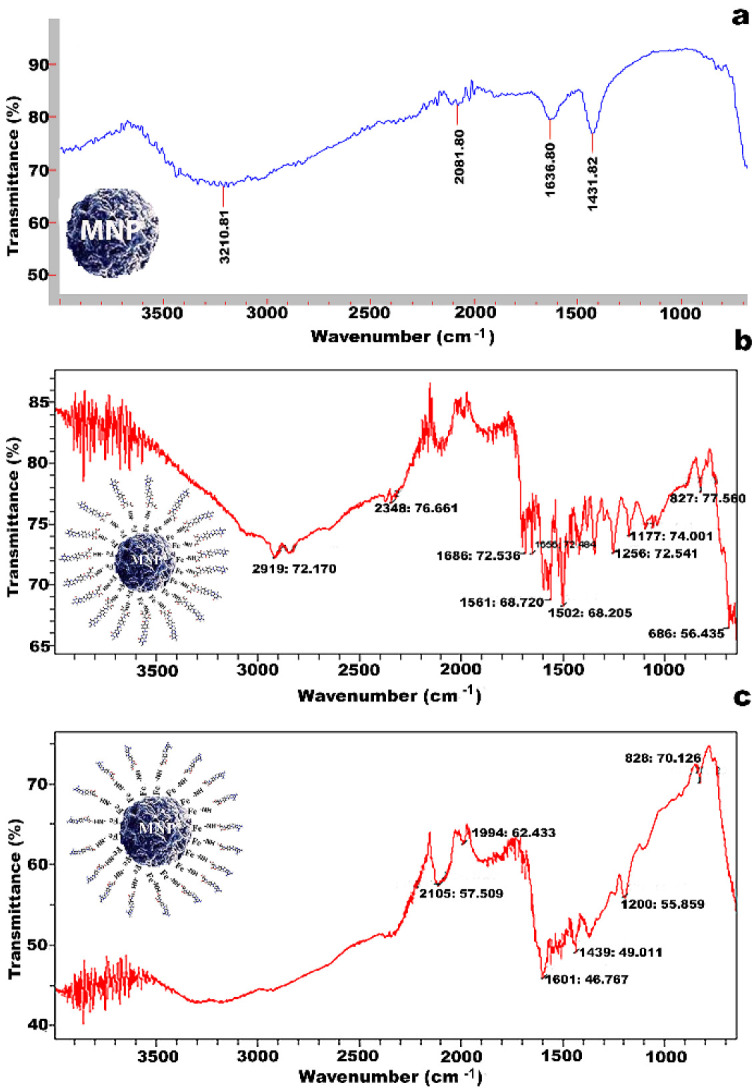
Models and FTIR spectra of MNPs with MTX and MNPs with folic acid. (**a**) FTIR spectrum shows the spinal structure of MNP with associated peaks. (**b**). FTIR spectra of MNP-MTX confirmed the conjugation of MTX on MNP. (**c**) Successful conjugation of MNPs with MTX.

The FTIR spinal structure of MNPs, as given in Figure 3a, showed associated characteristic peaks such as the Fe-O stretching band at 635.72 cm^−1^ due to stretching vibration mode Mt-O-Mo, H_2_O bending vibration at 1369.91 and amide group peak at 3424.80 cm^−1^. The FTIR spectrum of MNP-F (Figure 3b) showed that the streaked peak of NH_2_ and C=O groups of folic acid were observed at 2348 cm^−1^ and 1561 cm^−1^, respectively. The presence of a new peak at 1177 cm^−1^ confirmed the conjugation of the bond between –NH_2_ groups of MNPs and –COOH groups of folic acid by carbodiimide method. The FTIR spectra of MNP-MTX showed that the streaked peak of NH_2_ groups of MNPs was observed at 2105 cm^−1^. The presence of a new peak at 1439 cm^−1^ indicated the successful conjugation of the –NH_2_ groups of MNPs with the –COOH groups of MTX by carbodiimide method (Figure 3c). The models of conjugation of MNPs with folic acid and MTX are shown in Figure 1a,b, respectively.

### 3.3. Stoichiometry

A different preparation was used to calculate the binding capacity of MNP with folic acid and MTX. It was calculated that 0.02 gm contains 5.2 × 10^20^ to 5.5 × 10^20^ amine-terminated iron oxide nanoparticles bound with 1.14 × 10^20^ to 1.17 × 10^20^ molecules of folic acid and 1.24 × 10^20^ to 1.30 × 10^20^ MTX molecules, separately. Stoichiometry analysis was performed by standard calculations as given in Supplementary Data. Binding efficiency was calculated by taking the absorbance of pre and post-coupling solution and calculating percentage binding from the difference of absorbance. The binding efficiency of the anti-folate receptor antibody was almost 91% and that of MTX, almost 90% [48].

### 3.4. Characterization of Anti-Folate Receptor Antibodies

**Figure 4:**
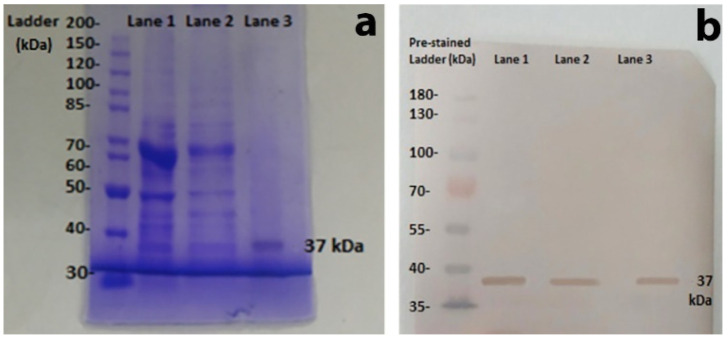
Western blot and SDS-PAGE results. (**a**) Purified FRs through SDS PAGE. Lane 1: crude sample containing sonicated HeLa cells; Lane 2: partial purification of folate receptor; Lane 3: magnetic affinity purified FR. (**b**) Western blot analysis of purified FR at 37 kDa molecular weight. Lane 1: crude sample containing crushed HeLa cells; Lane 2: partial purification of FR; Lane 3: magnetic affinity purified FR.

Anti-folate receptor antibodies were developed in rabbit by immunization with affinity purified folate receptor. ELISA was performed for the immunocharacterization of anti-FR antibodies, and results confirmed the presence of folate receptor antibodies. For molecular characterization, SDS PAGE was performed, and results showed the presence of FR protein at 37 kDa (Figure 4a). The mono-specific reactivity of anti-FR antibodies was further checked by Western blot analysis (Figure 4b). Samples were run on 10% SDS-PAGE and transferred onto nitrocellulose membrane. Immunochemical reaction showed clear bands at 37 kDa on the nitrocellulose membrane, which further confirmed the presence of an FR-specific antibody.

### 3.5. MagLISA

**Figure 5:**
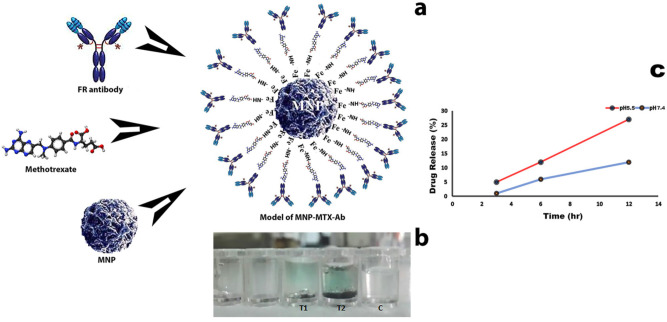
Schematic presentation, MagLISA and pH-dependent releasing behavior of MNP-MTX-FR Ab. (**a**) Schematic of conjugation of MTX on MNP and then FR antibody conjugation with nanocomposite on MTX. (**b**) MagLISA results. T_1_ and T_2_ are test wells, and C is the control well. (**c**) Drug releasing behavior at pH 5.5 and pH 7.4 at different time points.

Owing to the oxidizing property of the magnetic nanoparticles, MagLISA was performed, in which the blue color in the T_1_ and T_2_ wells indicated the presence of conjugated anti-FR antibodies and anti-MTX antibody in MNP-MTX conjugate. Control wells were devoid of reaction product (Figure 5b). The model of conjugation of MNP with MTX and anti-folate receptor antibody is shown in Figure 5a.

### 3.6. Nanocomposite Stability (In Vitro)

The release profile of MTX from conjugate after 12 h (in vitro) was determined at physiological and acidic pH. The drug was released slowly in basic buffer at pH 7.4 (12%) as compared to acidic buffer at pH 5.5 (27%), in vitro. Drug released from nanomedicines in tumor vicinity over a prolonged period of time. The tumor environment is acidic, and designed nanomedicine is pH-sensitive and releases faster at acidic pH as compared to the physiological pH of blood (Figure 5c).

### 3.7. Cellular Cytotoxicity of Different Nanocomposites

**Figure 6:**
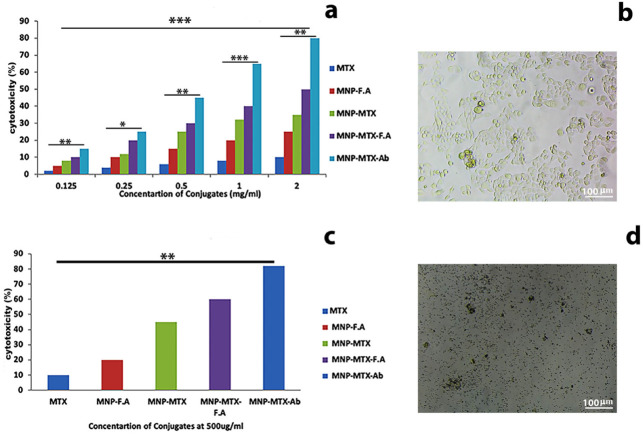
Cytotoxic effects of different conjugates to HeLa cells and MTT assay. Data represents in the form of mean (*n* = 3) ± SD with *p* value <0.0001 (***), <0.001 (**) and <0.01 (*). (**a**) Graph shows the percentage of cell cytotoxicity of different concentrations of nanocomposites. (**b**) Control (HeLa cells) showing cell growth in the absence of nanomedicines. (**c**) The cytotoxic effects through antibody-mediated endocytosis of different nanocomposites. (**d**) Test cells with conjugate MNP-MTX-FR Ab.

Cell cytotoxicity in both samples (control and test) were determined through MTT assay in the presence of composites and measured spectrophotometrically. In control, there was a significant amount of oxidoreductase activity in mitochondria, so high absorbance was recorded in live cells. On the other hand, test cells with different nanocomposites (MTT-treated cells) showed lesser absorbance due to cell death. Different conjugates were incubated with HeLa cells to observe their cellular cytotoxicity after 24 h. MNP-MTX-FR Ab nanomedicine exhibited maximum cellular cytotoxicity of 80% in 24 h, followed by MNP-MTX-FA with ~60% (Figure 6c). Data were in the confidence interval and showed the significance of comparison with *p* value < 0.001.

The cytotoxic effect of different nanocomposite concentrations increased proportionally with increasing conjugate concentration. The cells died due to the targeting of conjugate (MNP-MTX-FR Ab) to FR on HeLa cells by the endocytosis process (Figure 6a,d); on the other hand, control (Figure 6b) showed proper growth of cells in the absence of nanoconjugates. Means were compared with ANOVA and data in confidence interval with *p* value < 0.0001 for 1 mg/mL concentration, *p* value < 0.001 for 0.125, 0.5, and 2 mg/mL concentration, and *p* value < 0.01 for 0.25 mg/mL concentration.

### 3.8. Receptor Binding Assay of Nanomedicine with HeLa Cells

**Figure 7:**
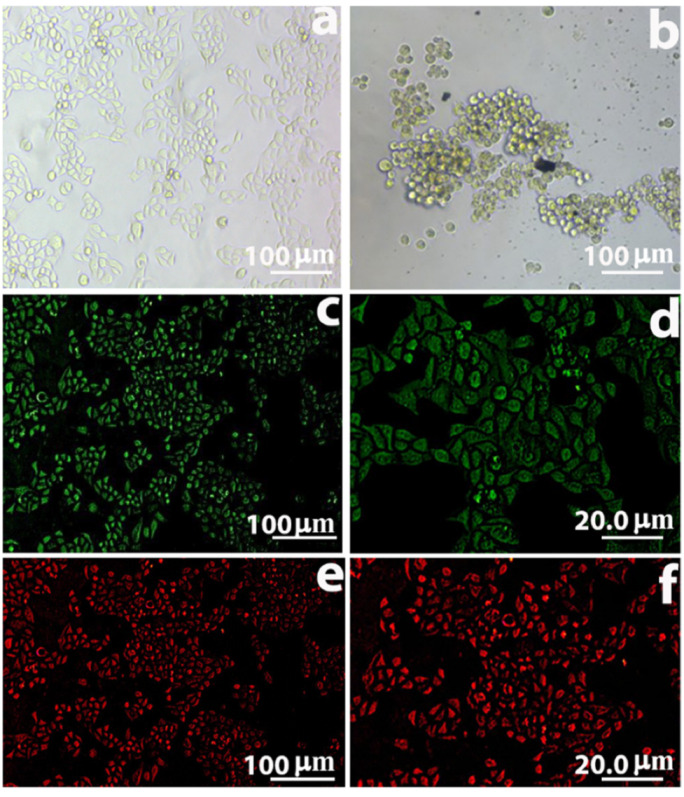
Binding assays–attachment of conjugate MNP-MTX-FR Ab to HeLa cells. Figure (**a**) Control. (**b**) Binding of conjugate MNP-MTX-FR Ab to HeLa cells. (**c**,**d**) Attachment of the FR antibody to HeLa cell’s folate receptor. (**e**,**f**) Attachment of MTX antibody to drug MTX.

Anti-FR antibody targeting of the FR on HeLa cells in binding assays is shown in Figure 7b. The presence of black color on cancer cells and not on empty spaces, shows the targeting of cells. The binding of MNP-MTX-FR Ab on the cell surface through FR targeting was confirmed by incubating HeLa cells with FITC conjugated anti-rabbit antibodies (Figure 7c,d). Green fluorescence on HeLa cells confirmed the successful attachment of nano formulations on HeLa cells. HeLa cells were incubated with anti-MTX antibody and then Texas red conjugated secondary antibody. Red fluorescence confirmed the presence of MTX in HeLa cells (Figure 7e,f). Green and red fluorescence shows the binding of the antibody though antibody-mediated targeting with the FR and MTX internalization.

### 3.9. Receptor Binding Assay of Nanomedicine with Tumor Tissue (Immunohistochemistry)

**Figure 8:**
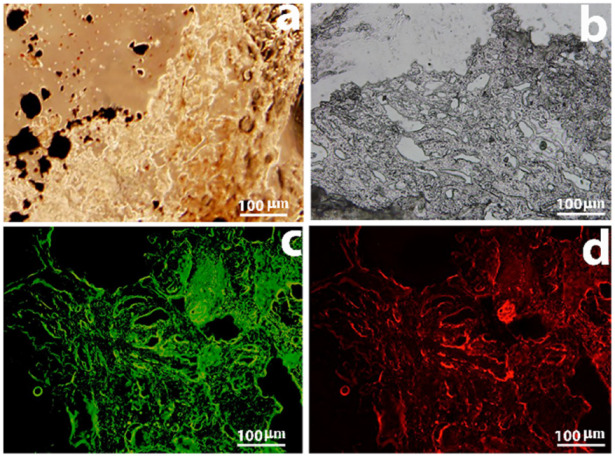
Conjugate MNP-MTX-FR Ab and tumor cell binding. (**a**) Tissue observation in bright field microscope. (**b**) Conjugate MNP-MTX-FR Ab and tumor section in phase contrast image. (**c**) Folate receptor antibody binding to folate receptors. (**d**) Attachment of anti-MTX antibody to MTX molecule on conjugate MNP-MTX-FR Ab.

Mouse mammary tumor was processed to examine the tissue-binding ability of nanocomposites. Binding and the presence of nanocomposites in tumor tissue were confirmed by examining the section under bright field microscope (Figure 8a,b); nanocomposites could be traced. Binding of FITC conjugated anti-rabbit antibodies to folate receptor antibody gave green fluorescence and confirmed (Figure 8c) the attachment of MNP-MTX-FA Ab nanocomposites to folate receptors expressed on tumor cells. Red fluorescence confirmed the attachment of anti-MTX antibody to MTX (Figure 8d), which subsequently attached to MNP-MTX-FR Ab, as discussed earlier.

## 4. Discussion

Scientists have been working on different targeted approaches for cancer cells to prevent the harmful effects of chemotherapeutic drugs on normal cells. Folate receptor expression increases thousands-fold on cancer cells compared to normal cells due to the increased demand for iron [42]. In addition, folate-based analogs work best for cancers overexpressing folate receptors, such as epithelial cancers including breast cancer, lung cancer, gastric cancer, gliomas, and female reproductive system carcinomas [49].

In this research project, a novel formulation of nanomedicine was designed to harness the synergistic effect of targeted drug delivery and immunotherapy. We examined the nanomedicine MNP-MTX-FR Ab for targeted drug delivery through folate receptors. Many targeted sites and combinations have been reported for targeted drug delivery, but a big gap still exists in the field of targeted nanomedicines.

In the current project, folate receptors were purified for the development of antifolate receptor antibodies. Receptors were purified by a novel method of magnetic affinity, designed in our Applied Molecular Biotechnology Research (AMBR) lab. HeLa cells express a high number of FRs for metabolic activity [50] because of their greater folic acid requirement for maintaining cellular functions [51]. HeLa cells were cultured to purify FR, using affinity purification by folic acid-linked MNPs. Purification of folate receptors from HeLa cells assured that the cancer cells expressed a form of folate receptor. Before using them for immunization, purified folate receptors were fully characterized by SDS PAGE, ELISA, and Western blot. SDS_PAGE size of ~37 KDa confirmed the purification of folate receptors [52]. Purified protein was used to immunize rabbit for the production of antifolate receptor antibody, and purified antibodies were further characterized by ELISA and Western blot before conjugation with MNP. ELISA and Western blot confirmed the development of anti-folate receptor antibodies with specific reactivity to folate receptors.

Magnetic nanoparticles were synthesized by hydrothermal coprecipitation, and their magnetic properties, size, shape, and surface chemistry were fully characterized by VSM, DLS, TEM, and FTIR, respectively. Synthesized magnetic nanoparticles have superparamagnetic properties, and their size falls into the range of 50–150 nm, which is necessary for their biomedical applications. The FTIR spectrum showed the specific spinal structure of MNP with associated peaks, specifically the Fe-O stretch at 650 cm−1 [53,54].

To synthesize the targeted nanomedicine, synthesized and fully characterized MNP was conjugated with MTX by the carbodiimide method, and conjugation was confirmed by FTIR analysis. The disappearance of old peaks from the spinal structure of MNP and the appearance of new peaks confirmed the conjugation. In targeted drug delivery, antibody-drug conjugates (ADCs) are also considered as an effective tool for targeting specific tumor antigens through antibody-mediated endocytosis [55], comprising antigen- or receptor-specific antibodies conjugated with the drug [56]. An anti-FR Ab was engineered onto nanocomposite (MNP-MTX) through carbodiimide linkage between the amino and the carboxylic group of MTX and anti-FR antibodies, respectively. Conjugation was confirmed by the FTIR study.

The presence of MNPs as carrier, drug, and anti-FR antibody in the final composite, MNP-MTX-FR Ab, was also confirmed by novel ultrasensitive colorimetric assay, magnetic nano enzyme-linked immunosorbent assay (MagLISA) [57], in which anti-MTX antibodies were coated on microtiter plate as a binder to detect the MTX. The MagLISA simultaneously detected the anti-MTX and anti-FR antibodies. Anti-MTX antibody was used to bind MTX in the conjugate MNP-MTX-FR Ab, while rabbit anti-FR antibody was allowed to bind with goat anti-rabbit HRP conjugated antibodies. The nanomedicine’s stability was examined at acidic pH 5.5 and at basic pH 7.4. The tumor environment is acidic, and the designed nanomedicine is pH-sensitive and releases faster at acidic pH compared to the physiological pH of blood. The nanomedicine is more stable in the initial hours at acidic and basic pH, and assures the release of the chemotherapeutic drug at the required site over a longer period of time. This property of nanomedicine is very important in in vivo applications.

The anti-cancer effect of synthesized nanomedicines was assessed against HeLa cells. An MTT assay was performed to check the cytotoxic effect of composites on cancer cells [58,59]. HeLa cells treated with conjugates indicated that control cells showed better NADPH absorbance activity than treated ones. [60]. Folic acid, a ligand of FR, undergoes upsurges in uptake molecules during high cellular biological activities [61,62,63]. Different conjugates were tested for cytotoxicity by the MTT assay in order to compare the effects of the novel MNP-MTX-FA Ab with other, previously reported nano formulations. Among different conjugates, such as MNP [64], MNP-FA [65,66], and MNP-MTX [67], MNP-MTX-FR Ab showed relatively high (80%) cytotoxicity through FR endocytosis.

Results showed that the final nano formulation successfully targeted the folate receptors and underwent receptor-mediated endocytosis of MTX. They further showed that the antibody for FR has more-specific affinity and gives better results as compared to folic acid. The anticancer potency of the conjugate nanomedicine was visualized through analysis of cell cytotoxicity towards cancer cell lines. MNP-MTX-FR Ab demonstrated maximum cytotoxicity, which confirms that antibody-coated medicine possesses potential affinity to destroy cancer cells in the targeted drug delivery process. Experiments are underway to assess the in vivo immunotherapeutic effects of this novel nanomedicine.

## 5. Conclusions

Our novel method involving magnetic nanoparticles functionalized with anti-folate receptor antibody and methotrexate (MNP-MTX-FR Ab) for antibody-mediated targeted drug delivery is efficient enough to eradicate tumor cells. Moreover, this approach is time-saving and will reduce the overall cost in nanocomposite formation, with minimal side effects to normal cells. This approach is more economical and feasible as compared to traditional chemotherapeutic methods, which require high doses of drug and also affect normal cell activities. The traditional drugs also increase cytotoxicity, cause immune suppression, require time for biodistribution, and are sometimes fatal.

## Data Availability

Not applicable.

## Supplementary Materials

The following supporting information can be downloaded online. Supplementary data: Calculation of Folic acid and Methotrexate bound to Magnetic Nanoparticles.

## Abbreviations

ADCs	Antibody-drug conjugates.
EDC	1-Ethyl-3-(3-dimethylaminopropyl) carbodiimide.
DHFR	Dihydrofolate reductase.
ECD	Ethyl carbodiimide.
ELISA	Enzyme linked immunosorbent assay.
FITC	Fluorescein Isothiocyanate.
FA-MNP	folic acid-MNP
FRs	Folic acid receptors.
FBS	Fetal bovine serum.
FR	Rabbit-anti Ab.
HRP	Horse radish peroxidase.
MNP-MTX-FR Ab	Anti-folate receptor antibody and methotrexate.
MTX	Methotrexate.
MTX-MNPs	MTX-conjugated nanoparticles.
MNPs	Magnetic nanoparticles.
NPs	Nanoparticles.
TMB	Tetramethylbenzidine.
