# Supplementary material for: A Novel Method of Magnetic Nanoparticles Functionalized with Anti-Folate Receptor Antibody and Methotrexate for Antibody Mediated Targeted Drug Delivery
Source: Molecules. 2022 Jan 1;27(1):261. doi: 10.3390/molecules27010261 (PMC8747068; doi:10.3390/molecules27010261)
Supplement: Supplementary file 1 [file molecules-27-00261-s001.zip › molecules-1528365-supplementary.pdf]

Production of anti-MTX antibody

Method:

2.4. Production and characterization of anti-MTX antibodies:

Six BALB/c mice of age 6 weeks were used for immunization to develop anti-MTX antibodies. Before starting the immunization process, preimmune blood was drawn from mice tails to be used as a control. The BSA (bovine serum albumin) and ovalbumin (OVA) were conjugated with MTX for immunization purpose. BSA (20 mg/ml) or OVA (20 mg/ml) was dissolved in 10 ml of 0.1 M sodium phosphate buffer with a pH of 6.8 separately. MTX was conjugated with BSA and oval albumin separately for developing and screening antibodies against MTX, respectively. Glutaraldehyde was added to a final concentration of 1.25% in each tube. The mixture was kept overnight with gentle stirring at 25°C and then, was dialyzed against PBS to remove excess glutaraldehyde from activated BSA or OVA. Then, 10 ml of MTX (10 mg/ml) of 0.5 M sodium carbonate buffer with a pH of 9.5 was added into activated BSA or OVA and kept overnight at 4°C followed by the addition of 0.5ml of 200 mM glutaraldehyde into mixture (at room temperature for 2 hours) with constant agitation. Mice were immunized with BSA-MTX conjugate (80μg conjugates in PBS/time) for two months at ten-day intervals. The serum was separated from immunized blood and stored at −20°C. Antibody was characterized by ELISA with 100μl of OVA-MTX (100μg conjugates) was coated in microtiter wells (triplicate) and followed the same procedure as described above (Lodhi & Samra, 2020).

Results

3.5. Development of anti-MTX antibody and characterization:

For immunization, MTX was conjugated with oval albumin and BSA through glutaraldehyde method. Mouse anti-MTX antibodies were developed in mice by immunization with BSA-MTX. The presence of antibodies in serum was checked by using OVA-MTX as screening antigen. The development of blue color indicated the specificity of the antibodies.

Discussion:

Anti-MTX antibodies were also developed and characterized to track the MTX conjugated nanomedicines in cancer cells and tissue. A cysteine-specific MTX linkage to BSA yields profound efficacy glioma cell line (Cooper et al., 2016). We conjugated MTX with BSA to develop the antibodies and its specific binding was characterized by ELISA using another MTX-OVA complex. Further, the specificity of the anti-MTX antibody was also confirmed by MagLISA. For MagLISA assay, MTX was conjugated with MNP separately and the development of a blue-colored complex explains the oxidizing properties of MNPs with the substrate, which, in turn, showed the presence of MTX in nanocomposites.

Supplementary Data

Calculation of Folic acid and Methotrexate bound to Magnetic Nanoparticles:

Molecular weight of MNPs = 231.53g

Molecular weight of Folic acid = 441.4g

Molecular Weight of Methotrexate = 454.44g

Avogadro's number = 6.02×10^{23}

1mole of MNP = 231.53g

1 mole = Avogadro's number

231.53g of MNP contains 6.02×10^{23} molecules

0.02g of MNP contain molecules = $\frac{0.02\times6.02\times10^{23}}{231.53} = 5.201\times10^{20}$

This means that 0.02 g of MNPs contain 5.201×10^{20} molecules

4.1.4 Binding of Folic acid (100mg) with MNP:

Pre-coupling A_{460} = 0.874

Post-coupling A_{460} = 0.801

0.874 for 100mg

0.801 for $\frac{0.801\times100}{0.874}$ = 91.647 (Binding efficiency %)

100 – 91.47 = 8.35mg = 0.00835g

441.4g folic acid contains molecules = 6.02×10^{23}

0.00835g folic acid contains molecules = $\underline{0.00835\times6.02\times10^{23}} = 1.139\times10^{20}$

441.4

This means that 1.139×10^{20} molecules of Folic acid were attached to 5.201×10^{20} molecules of MNPs.

4.1.5 Binding of Methotrexate (100mg) with MNPs:

Pre-coupling A_{300} = 0.811

Post-coupling A_{300} = 0.735

0.811 for 100 mg

0.735 for $\frac{0.735\times100}{0.811}$ = 90.62 (Binding efficiency %)

100-90.62 = 9.37mg = 0.00937g

454.44g of Methotrexate contain molecules =

0.00937g Methotrexate contain molecules = $\frac{0.00937\times6.02\times10^{23}}{454.44} = 1.241\times10^{20}$

This means that 1.241×10^{20} molecules of Methotrexate were attached to 5.201×10^{20} molecules of MNPs.
